# In vitro validation concept for lyophilized fecal microbiota products with a focus on bacterial viability

**DOI:** 10.1007/s11274-025-04291-0

**Published:** 2025-02-27

**Authors:** Sara A. Sedeek, Fedja Farowski, Stella Youssafi, Anastasia Tsakmaklis, Susanne Brodesser, Madiha M. El-Attar, Mohamed Omar Abdelmalek, Maria J. G. T. Vehreschild

**Affiliations:** 1 Department of Internal Medicine II, Infectious Diseases, Goethe University Frankfurt, University Hospital Frankfurt, Frankfurt am Main, Germany; 2 Department of Tropical Medicine and Gastroenterology, Assiut University, Assiut, Egypt; 3 Faculty of Medicine, Department I of Internal Medicine, Centre for Integrated Oncology Aachen Bonn Cologne Duesseldorf, University of Cologne, University Hospital Cologne, Cologne, Germany; 4 German Centre for Infection Research (DZIF), Partner Site Bonn-Cologne, Cologne, Germany; 5 Faculty of Medicine, Cluster of Excellence Cellular Stress Responses in Aging-associated Diseases (CECAD), University of Cologne, University Hospital of Cologne, Cologne, Germany

**Keywords:** Bacterial viability, Capsules, *Clostridioides difficile*, Fecal microbiota transplantation, Lyophilization

## Abstract

Fecal microbiota transplantation (FMT) effectively treats recurrent *Clostridioides difficile* infection (rCDI), typically administered as a fresh or frozen stool suspension through colonoscopy, nasojejunal tube, or oral capsules. Lyophilized fecal microbiota (LFM) are an alternative to frozen FM products. We aimed to assess whether lyophilization affects bacterial viability and metabolite levels and to develop LFM capsules for clinical use in Germany. Fecal donations from pre-screened volunteers were aliquoted and analyzed through microbial cell counting, bacterial culture, 16S rRNA gene amplicon sequencing, and bile acid assays. Results showed higher counts of viable bacterial cells and cultured anaerobes in unprocessed stool compared to freshly processed stool (*p* = 0.012 and *p* < 0.001, respectively). No significant difference in viable bacterial counts was found between freshly processed (day 0), lyophilized (day 3) and frozen FM (day 3) (*p* = 0.15), nor between freshly processed (day 0), lyophilized (days 30 and 90) and frozen FM (day 30) (*p* = 0.07). lyophilization did not significantly impact bile acid and 16S rRNA profiling. Encapsulation of lyophilized powder required fewer capsules (10–14) than frozen capsules (30). LFM products are a practical, viable alternative to frozen and fresh FM products, potentially improving storage and patient acceptance.

**Supplementary Information:**

The online version contains supplementary material available at 10.1007/s11274-025-04291-0.

## Introduction

*Clostridioides difficile* (*C. diff*), previously known as *Clostridium difficile*, is the most common cause of healthcare-associated infectious diarrhea (Elliott et al. 2017; Czepiel et al. 2021). While most initial episodes can be treated successfully with fidaxomicin or vancomycin, recurrent disease affecting a total of 15–20% of *Clostridioides difficile* infection (CDI) patients remains a major challenge in clinical practice (van Prehn et al. 2021).

Fecal microbiota transplantation (FMT) is defined as the transfer of a healthy donor’s gut microbiota into the gut of a patient in an attempt to restore physiological microbiota diversity and function (Halaweish et al. 2022). It is increasingly employed to treat rCDI when antibiotics are ineffective (van Nood et al. 2013; Baunwall et al. 2021), with reported efficacy up to 90% (Halaweish et al. 2022). Hence, most national and international guidelines advocate using FMT as a secondary prophylaxis to prevent a next episode in multiply rCDI (Kelly et al. 2021; van Prehn et al. 2021; Stallmach et al. 2023). In addition, the therapeutic impact of FMT on a broad range of diseases beyond rCDI, including metabolic, malignant, autoimmune, and neuro-psychiatric diseases is being assessed in clinical trials (Halaweish et al. 2022).

After the first randomized controlled clinical trial on the use of FMT was published in 2013 (van Nood et al. 2013), FMT was typically delivered as a freshly processed suspension via colonoscopy, enemas, a nasogastric or a nasoduodenal tube (Kim and Gluck 2019). The introduction of frozen products substantially facilitated the logistics of FMT and thus broadened patient access. FMT products stored at -80 °C are now routinely used, and multiple randomized controlled trials have proven that it is as effective as fresh suspensions in terms of rCDI cure rates (Youngster et al. 2014; Lee et al. 2016; Gangwani et al. 2023). Successful encapsulation of FM represents another important procedural improvement with respect to patient acceptance, associated procedural risks, as well as expenses (Kao et al. 2017; Ramai et al. 2021; Halaweish et al. 2022).

While frozen encapsulated fecal microbiota (FM) offers substantial logistic advantages compared to fresh FMT products, storage, stability and transportation of these products still relies on an expensive infrastructure. More stable formulations are needed to optimize usability for physicians and patients alike. Lyophilization or freeze-drying represents the optimal method for preservation in this context (Khoruts et al. 2021).

Lyophilization is a two-stage process that involves rapid deep-freezing of the stool suspension followed by the sublimation of water under reduced pressure. As a result, the fecal suspension turns into a lyophilized cake that is ground into a powder with reduced volume, which in turn may reduce the number of capsules, required for one treatment unit; moreover, storage and transport costs can be reduced and the powder has a less offensive smell, improving patient acceptance (Staley et al. 2017; Reygner et al. 2020; Varga et al. 2021; Zain et al. 2022). These benefits are, however, potentially offset by concerns that the processing might threaten microbial viability. In many countries, FM products are regulated as medicinal products and are therefore subject to Good Manufacturing Practice (GMP) requirements, if to be used in clinical trials. In this context, a complete validation of the manufacturing process is needed. Such a plan would also have to address the above-mentioned viability issues. With this work, we want to present a complete approach to a GMP-compatible validation of LFM products in comparison to fresh and frozen products as the current gold standard with a special focus on bacterial viability.

## Materials and methods

### General concept

**Fig. 1:**
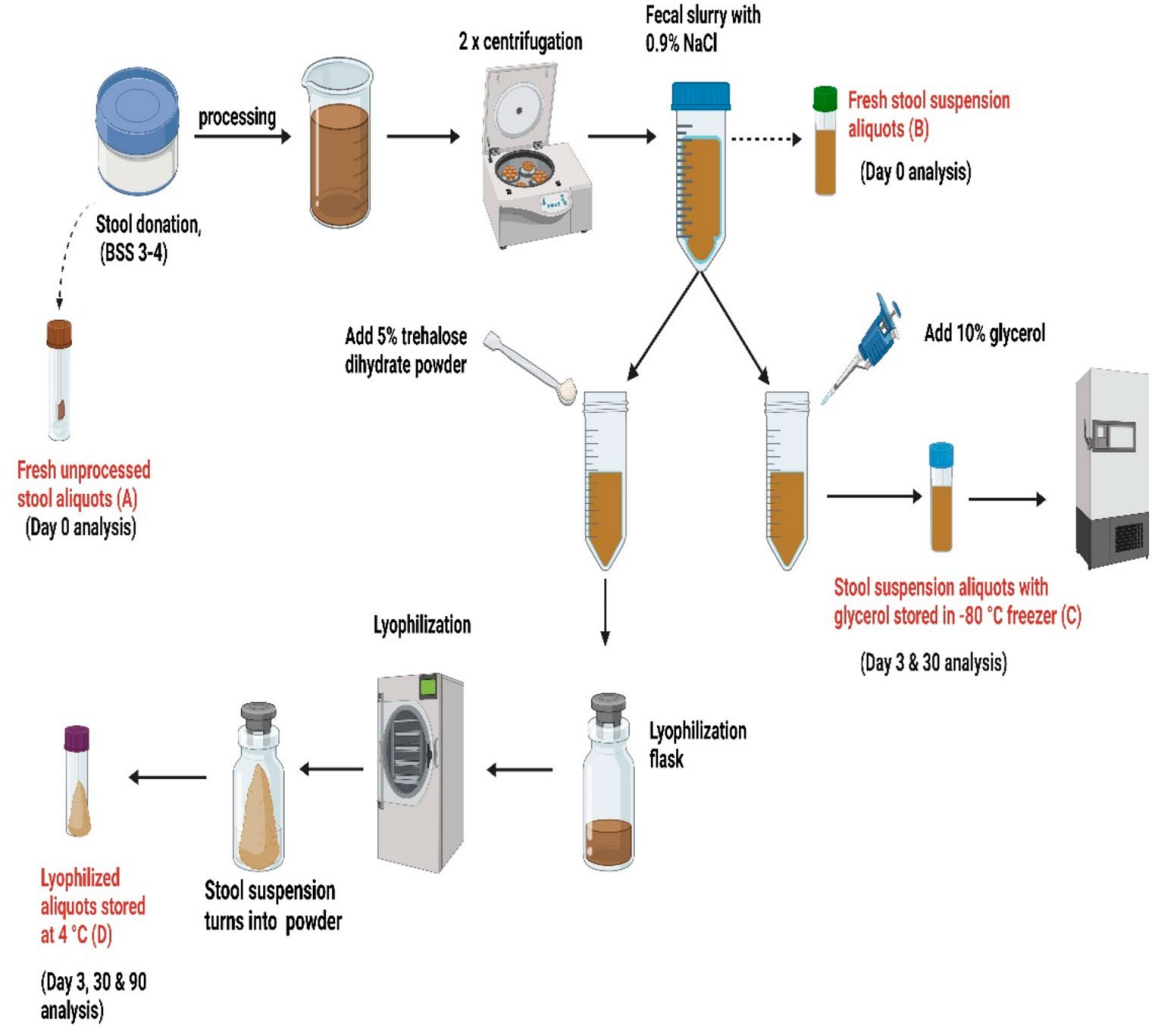
Processing of donor stool. Microbiological analysis of the fresh stool was performed on day 0; frozen and lyophilized samples were assessed on day 3 and day 30. Additional analysis of lyophilized samples using the microbial cell counter was performed on day 90. BSS: Bristol stool scale. Image created inBioRender. Biehl, L. (2025) https://BioRender.com/u48x603

The overall validation approach is shown in Fig. 1. Eight healthy pre-screened donors (Supplementary material, Table S1) were chosen using the Cologne Microbiota Bank (CMB) questionnaire, and in accordance with the standardised model for stool banking for FM products (Keller et al. 2021). Each individual provided one stool donation with a minimum weight of 120 g that was processed within half an hour after donation in the GMP facility of the CMB.

From each donation, we examined the bacterial viability of four formulations, i.e. fresh unprocessed stool, fresh processed stool suspension, frozen stool suspension and lyophilized stool suspension at designated time points. We used the QUANTOM Tx™ microbial cell counter and bacterial culture (for aerobes, anaerobes and spores) to analyze microbial viability. Bacterial 16S rRNA gene profiling and bile acids analysis were used to further specify potential changes in the content of the LFM product. Residual water content was determined using the volumetric Karl-Fischer Titration.

### Fecal processing

#### Initial processing and aliquoting

Of every donation, 5 g of stool were used for the analysis of fresh unprocessed stool. Then, we homogenized 112 g of stool from the remaining donation with 500 ml of NaCl 0.9% under aerobic conditions directly in a stomacher bag equipped with a filter, allowing for removal of large food particles and fibrous material. We split the filtrate into twelve falcon tubes (50 mL, each), that were centrifuged at 400xg for 5 min for further separation of undesirable suspended matter. The resulting pellet was discarded. The supernatant was submitted to a second centrifugation step at 6000xg for 10 min. After the second centrifugation, the resulting supernatants from the falcon tubes were discarded and the pellets were combined to produce a fecal slurry (40 mL). A total of 6 mL was used for analysis of the fresh stool suspension (without cryoprotectants, Fig. 1). Half of the remaining fecal slurry was utilised for the preparation of a frozen aliquot by the addition of glycerol 85% (Ph. Eur. 250 ml, Otto Fischer GmbH & Co. KG, 27310) as a cryoprotectant before freezing to achieve a final concentration of 10% *v/v*. For the lyophilized product, we added D-(+)-trehalose dihdrate powder (Sigma-Aldrich, 90210, Germany) to the other half as a lyoprotectant before lyophilization to achieve a final concentration of 5% *w/v*.

Each sample type was split into several aliquots for microbiological analysis, all originating from the same amount of starting material (the same concentration of stool/ml). We divided the lyophilized sample into multiple aliquots; the weight of each was measured separately for each donor batch directly after lyophilization. The fresh stool suspensions (unprocessed and processed) were analyzed on day 0, while the frozen and lyophilized suspensions were analyzed twice: on day 3 and day 30, after freezing and lyophilization, respectively. We additionally studied bacterial viability of the lyophilized suspension of a subset of 5 stool donors on day 90 using the QUANTOM Tx™ microbial cell counter.

#### Lyophilization

To build our lyophilization methodology, we performed a pilot study at Martin Christ GmbH, Osterode am Harz, Germany, where lyophilization of fecal suspensions with trehalose of 3 samples was performed using the EPSILON 2–4 LSC plus pilot freeze-dryer (Martin Christ GmbH, Germany). Primary and secondary drying lasted 50 h and 8 h, respectively, using a shelf temperature of -23 °C and under 0.180 mbar vacuum and of + 20 °C and under 0.05 mbar vacuum, respectively. The lyophilized samples were aliquoted, vacuum-sealed and stored at 4 °C. Based on the results of the pilot study (microbial cell counter and bacterial culture), we adapted our lyophilization approach and set up a less intensive lyophilization protocol at the CMB, Cologne, Germany using the same freeze-dryer model for the processing of the 5 remaining samples. Primary and secondary drying was set at 35 h and 2 h, respectively. All other parameters remained unchanged.

After lyophilization, we determined the volume of sublimated water for each lyophilized stool patch (weight of stool suspension before lyophilization - weight of lyophilized stool cake after lyophilization), in order to allow for subsequent resuspension, which is necessary to allow for microbiological analysis.

### Microbiological analyses

#### Evaluation of bacterial viability

We assessed the total and viable bacterial cell count of each sample preparation using the QUANTOM Tx™ microbial cell counter (Logos Biosystems, Ltd., Korea, Q10001), which is a novel image-based, automated cell counter, that can quantitively measure fluorescence-stained bacterial cells in a solution in a few minutes (Bainbridge-Sedivy et al. 2023), unlike flow cytometers, which are faster and more accurate, but more expensive and technically demanding. Ten images of fluorescence-stained cells are captured and analyzed automatically for rapid and accurate bacterial cell counts, in even the tightest clusters, and an example of a cell count report is provided in the supplementary material.

Each sample was serially diluted in duplicates (1:200, 1:400, 1:800 and 1:1600); for each dilution, we examined the total bacterial count by mixing a 10 µL sample with 1 µL of QUANTOM™ total cell staining dye (Q13101) and 1 µL of QUANTOM™ Total Cell Staining Enhancer (Q13002) and then incubated for 10 min at room temperature. For the viable cell count, a 10 µL sample was mixed with 2 µL of QUANTOM™ viable cell staining dye (Q13201) and then incubated for 30 min at 37°C. The stained bacterial cells (either total or viable dye) were then mixed with 8 µL QUANTOM™ Cell Loading Buffer I (Q13001), loaded onto QUANTOM™ M50 Cell Counting Slides, and centrifuged at 300xg for 10 min in the QUANTOMTx™ Centrifuge. The slides were thereafter inserted into the QUANTOM Tx™ slide port, and results of the bacterial cell counts were expressed as bacterial cells/ml and recalculated as bacterial cells/g initial stool. The samples were analyzed using the QUANTOM Tx microbial cell counter using the following parameters: size gating 0.3 to 50 μm; roundness 30%; declustering level 7; detection sensitivity 4. In both total and viable cell counts, we used the average of the results of the following dilutions (1:400, 1:800 and sometimes 1:1600), and the bacterial count in each dilution was measured twice.

Subsequently, we wanted to determine the viability of the bacteria within the lyophilized stool suspensions after different stages of lyophilization to assess, if secondary drying may impact bacterial viability. To that aim, we utilized 6 other different stool donations. The viability in each donation was measured in triplicates at different points during the lyophilization process, including at the end of primary drying and at the end of secondary drying. We also used those 6 preparations to measure the amount of residual water after primary and secondary drying.

#### Bacterial culture

One aliquot of each preparation was serially diluted (1:200, 1:400, 1:800 and 1:1600) with saline solution (NaCl 0.9%) in duplicates. From each dilution, 50 µL were inoculated on 5% sheep blood agar using an Eddy Jet 2 W automatic spiral plater (IUL, Cat. 90003800, Barcelona, Spain). The cultures were incubated for 24 h at 37 °C under aerobic conditions.

For anaerobes, Schaedler agar plates were used. After inoculation, the plates were placed in anaerobic jars (the Anoxomat ^®^ III anaerobic Jar system) and incubated for 48 h at 37 °C. Anaerobic conditions were confirmed by an indicator. For culturing spores, 15 µL stool suspension was diluted (1:10 and 1:100) with ethanol 99% and then plated on Schaedler agar plates and incubated for 48 h at 37 °C under anaerobic conditions. The amount of colony forming units (CFU/ml) was counted using the SphereFlash^®^ automatic colony counter (IUL, Cat. 90007000, Barcelona, Spain) and expressed as CFU/g initial stool.

#### Bile acid analysis

This analysis was conducted at the CECAD Lipidomics/Metabolomics Facility in Cologne using the AbsoluteIDQ^®^ Bile Acids kit (Biocrates, Innsbruck, Austria) for quantification of primary and secondary bile acids by liquid chromatography tandem mass spectrometry (LC-MS/MS) (Pham et al. 2016). The kit includes external and internal calibration standards and quality control samples (QC). Bile acids are quantified using seven levels of external calibration standards (µmol/L) and 10 stable isotope-labeled internal standards. By correcting matrix effects and evaporation during sample preparation, the internal standards are added to the external calibration standards and the samples to guarantee the quality of the measurements. The quality control samples (QC) are based on human serum and consist of three different spike levels: low (QC1), medium (QC2), and high (QC3). The acceptable variation range of measured concentrations in QC2 and QC3 is +/- 30%, in QC1 +/- 45%. We used the MultiQuant 3.0.3 software (AB SCIEX) to integrate the peaks of the isotope-labeled and non-labeled (endogenous) bile acids in the LC-chromatograms, compute calibration curves, and quantify the endogenous bile acids based on the calibration curves (Pham et al. 2016). We always used 200 mg of the unprocessed stool and 200 µl of the stool suspension or the reconstituted lyophilized stool suspension in addition to 600 µl extraction buffer for bile acids extraction. Since the aliquots were frozen at -80 °C until the moment of bile acid extraction, this measurement was not performed on fresh stool. We measured six primary/conjugated primary bile acids (cholic acid, taurocholic acid, glycocholic acid, chenodeoxycholic acid, taurochenodeoxycholic acid, and glycochenodeoxycholic acid), as well as eight secondary or conjugated secondary bile acids (ursodeoxycholic acid, glycoursodeoxycholic acid, deoxycholic acid, taurodeoxycholic acid, glycodeoxycholic acid, lithocholic acid, taurolithocholic acid, and glycolithocholic acid) in aliquots of four stool donations in duplicates; results were expressed as pmol/g.

#### Microbiota profiling by 16S rRNA gene amplicon sequencing

The genomic DNA from each sample was extracted using the FastDNA™ SPIN Kit for soil (MP Biomedicals, Solon, OH, USA) and used for taxonomic profiling by amplicon sequencing of the V3-V4 region of the bacterial 16S rRNA gene on the Illumina MiSeq (illumina 2013; Klindworth et al. 2013). Sequencing data were processed using the DADA2 pipeline and QIIME version 2 (release 2021.8) (Caporaso et al. 2010; Callahan et al. 2016). Taxonomic classification was performed using a Naïve Bayes classifier (sklearn v0.24.1) (Pedregosa et al. 2011), trained on SILVA database release 138 (Quast et al. 2012). Microbiota analyses were carried out using R for Statistical Computing (version 4.2.0, R Foundation for Statistical Computing, Vienna, Austria) (R Core Team 2018). Alpha diversity and beta diversity metrics of the different sample preparation methods were calculated using the R package phyloseq (1.48.0) (McMurdie and Holmes 2013). The beta diversity, in this case the Bray-Curtis distances between the samples, were visualized using principal coordinate analysis (PCoA) and a hierarchical cluster analysis; the effect of the preparations on the beta diversity was tested by a permutational multivariate analysis of variance (PERMANOVA).

#### Residual water measurement

We measured the residual water of six lyophilized preparations. Each donation was measured in triplicates. The residual water was measured at two time points (at the end of primary and secondary drying) according to the manufacturer’s instructions (Honeywell, United States, Charlotte). Initially, the water content of Solvent E was assessed. Then 10 ml of Solvent E were added to each of the lyophilized aliquots after primary and secondary drying. Titrant 5E was added using a 1 ml Hamilton syringe. The titrant solution was added gradually, and the suspension was continuously stirred by a magnetic stirring rod.

A yellow colour shift indicated the point at which all water contained in the sample had been consumed by the reaction. The consumption of titrant was read of the syringe, and the water content was calculated from the amount of titrant used. The previously calculated water content of Solvent E was subtracted from the total water content. For each donation, a triple determination was carried out for both the primary drying and the secondary drying step.

### Statistical analysis

Statistical analysis of the results from the QUANTOM Tx™ microbial cell counter, the bacterial culture, and bile acid assay was done using GraphPad Prism 8.0.1, for Windows (GraphPad Software, San Diego, California USA). Data were assessed for normality of distribution using the Shapiro-Wilk and Kolmogorov-Smirnov tests.

For normally distributed data, results were presented as mean and standard deviation (SD); Paired sample t-test was used to compare the mean of two samples; and repeated measures one-way ANOVA with post-hoc Tukey HSD test was used to compare the mean of three or more groups. For non-normally distributed data, results were presented as median and range; Wilcoxon signed rank test was used to compare the mean of two samples; and Friedman test with Dunn’s multiple comparisons was used for three or more groups. All statistical tests were two-tailed, and a *p*-value < 0.05 was considered statistically significant.

## Results

Data from the pilot study are summarized in the supplementary material together with data from the main study (Tables S2-S6). Data from the main study (5 donor stool samples) is presented in detail here.

### Total and viable bacterial cell counts using the QUANTOM Tx™ microbial cell counter

#### Total bacterial cell count

**Fig. 2:**
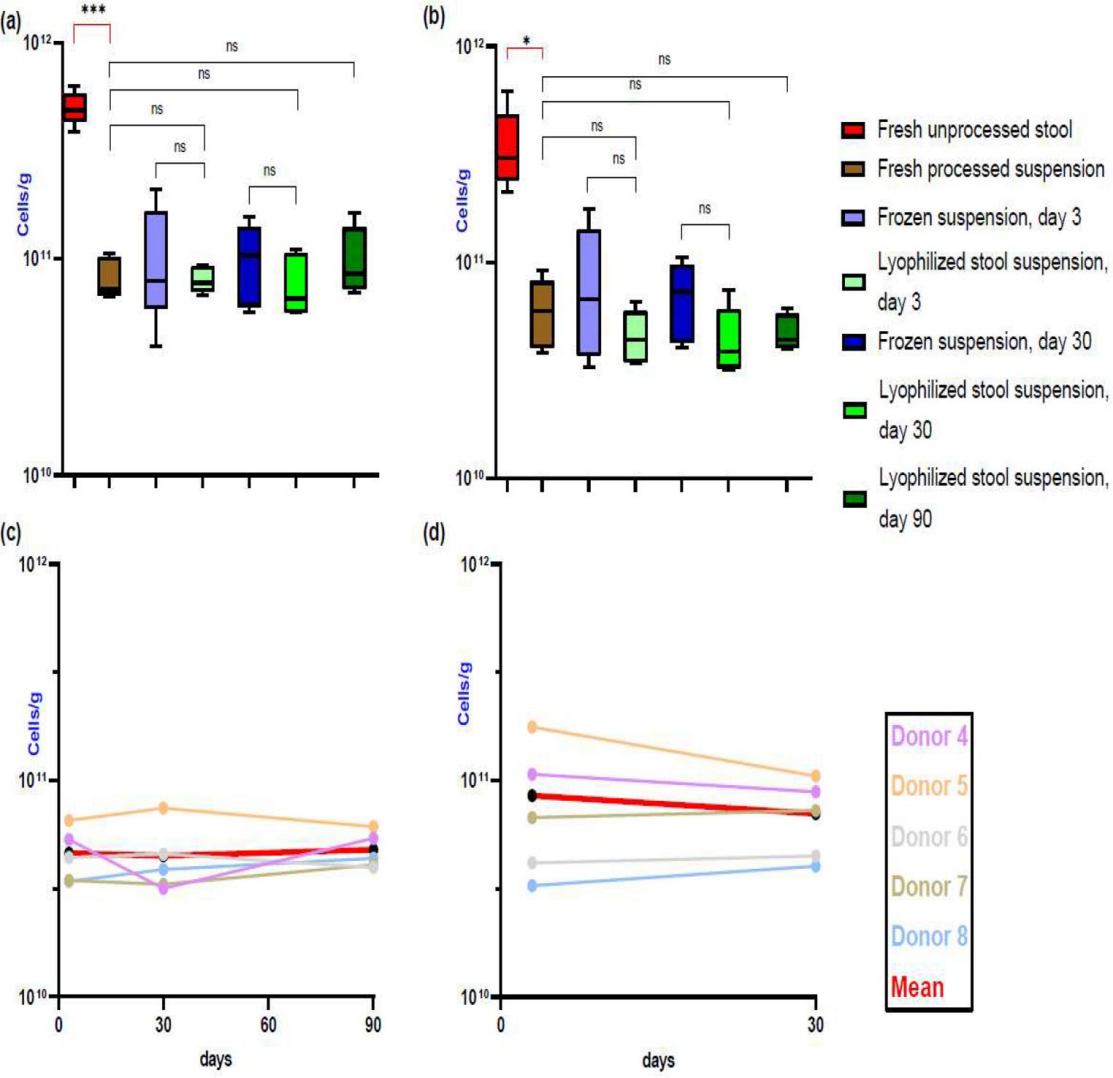
Results of the QUANTOM Tx^™^ microbial cell counter were observed for different fecal microbiota preparations and time points: (**a**) total bacterial cell concentration (*n* = 5), (**b**) viable bacterial cell concentration (*n* = 5), (**c**) viable cell concentration of the lyophilized stool suspension on day 3, 30 and 90 (*n* = 5), and (**d**) viable cell concentration of the frozen suspension on day 3 and 30 (*n* = 5). Results are presented as mean ± SD. * *p* < 0.05, **p < 0.01, ***p < 0.001

In the fresh unprocessed stool, the total bacterial cell concentration (both living and dead cells) was 5.02 × 10^11^ cells/g, and it reached 8.23 × 10^10^ cells/g in the freshly prepared suspension, which corresponds to a recovery yield of 16,4%. The mean of the total cell concentration in the resuspended lyophilized samples on day 3 was 8.05 × 10^10^ cells/g. There were no significant differences in cell concentrations for all follow-up samples (Fig. 2a). Furthermore, there was no significant difference in cell counts after secondary drying and primary drying (Supplementary material, Table S7 and Fig. S1).

#### Viable bacterial cell count

In the fresh unprocessed stool, the viable bacterial cell concentration was 3.5 × 10^11^ cells/g and reached 6.07 × 10^10^ cells/g in the freshly prepared suspension, which corresponds to a recovery yield of 17.33%. The mean of the viable bacterial cell concentration in the resuspended lyophilized samples on day 3 was 4.62 × 10^10^ cells/g. There were no significant differences in the viable cell counts of all follow-up samples (Fig. 2b, c and d). After one month at -80 °C, the percent of viable bacterial cells in the frozen suspensions was 69%, while after one month at 4 °C, the percent of viable cells in the lyophilized stool suspensions was 57% (Supplementary material, Table S8). When investigating the viable cell count during lyophilization stages, differences between different time points were statistically not significant (Supplementary material, Table S9 and Fig. S1). The viable cell concentration was approximately 60% at the start of lyophilization and remained above 55%, suggesting at least 55% of the total cells remained intact throughout the process.

Total and viable bacterial cell concentration measurements (cells/g) of the different microbiota preparations are shown in the supplementary material (Table S2 and S3).

### Bacterial culture

**Fig. 3:**
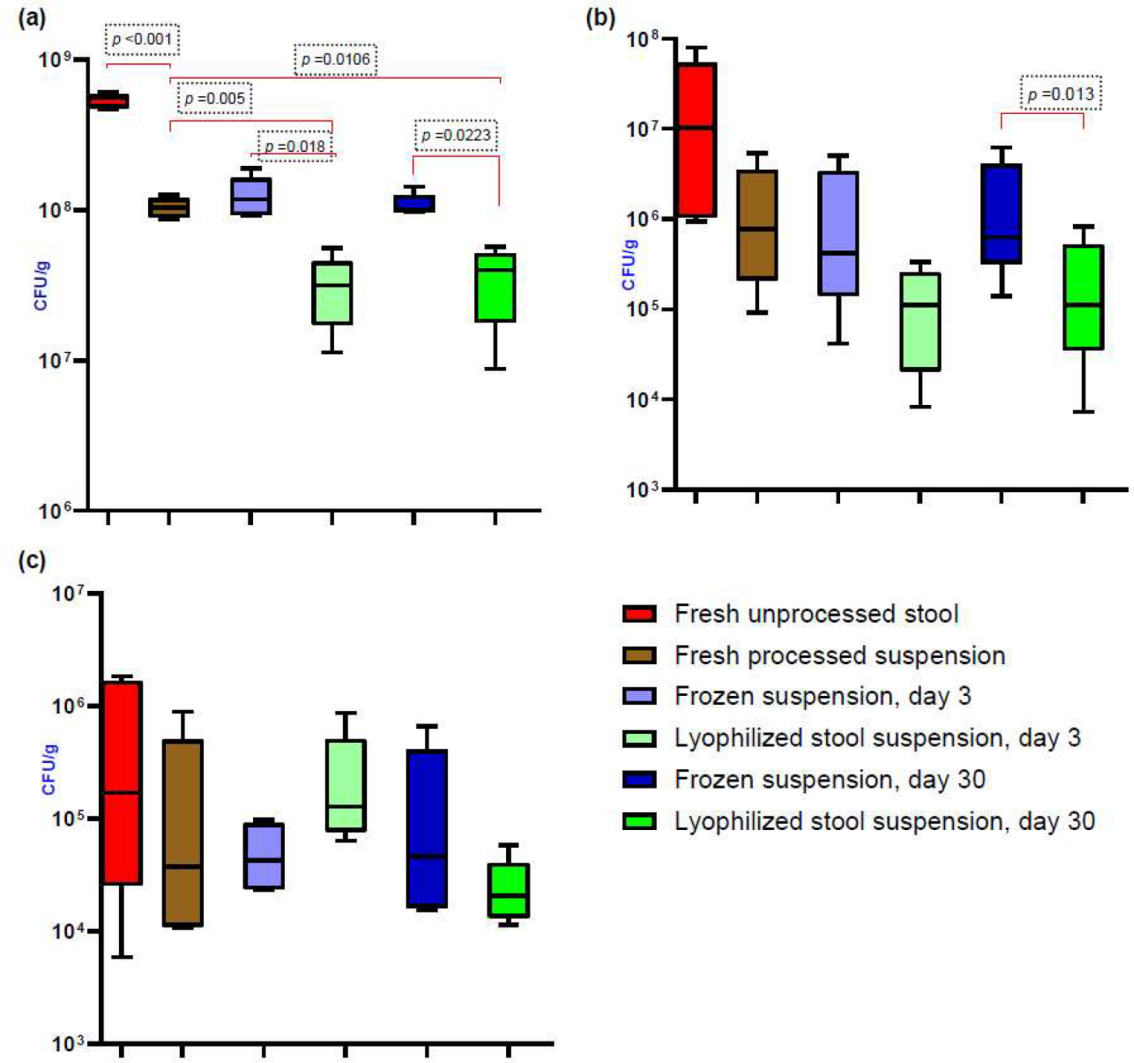
Results of bacterial culture (CFU/g) for the different fecal microbiota preparations. (**a**) culture of anaerobes, showing that the mean CFU/g in lyophilized stool suspension (D3) is significantly lower than in frozen suspension (D3) and fresh suspension (D0), and that the mean CFU/g in lyophilized stool suspension (D30) is significantly lower than in frozen suspension (D30) and fresh suspension (D0) (**b**) culture of aerobes, where the median CFU/g in lyophilized stool suspension (D30) is significantly lower than in frozen suspension (D30) and (**c**) culture of spores with no statistically significant differences between preparations. Results are expressed as mean ± SD or median and range

Stool preparations were cultured for anaerobes, aerobes and spores (Fig. 3). No statistically significant differences were observed between the lyophilized stool suspension on day 3 and 30 and between the frozen suspension on day 3 and 30 (Supplementary material, Fig. S2).

Bacterial culture results of the different microbiota preparations (CFU/g) are shown in the supplementary material (Table S4-S6).

### Bile acids analysis

Bile acid analyses were carried out in four out of the five stool donors’ FM preparations in duplicates (unprocessed stool, processed stool suspension without glycerol, processed stool suspension with glycerol and lyophilized stool suspension), as shown in Table S10.

Five of the measured secondary bile acids were identified in all donors’ aliquots, but taurolithocholic acid and glycoursodeoxycholic acid were detected in only two donors’ samples, while glycolithocholic acid was detectable in only one donor’s sample. Therefore, we excluded them from the analysis.

**Fig. 4:**
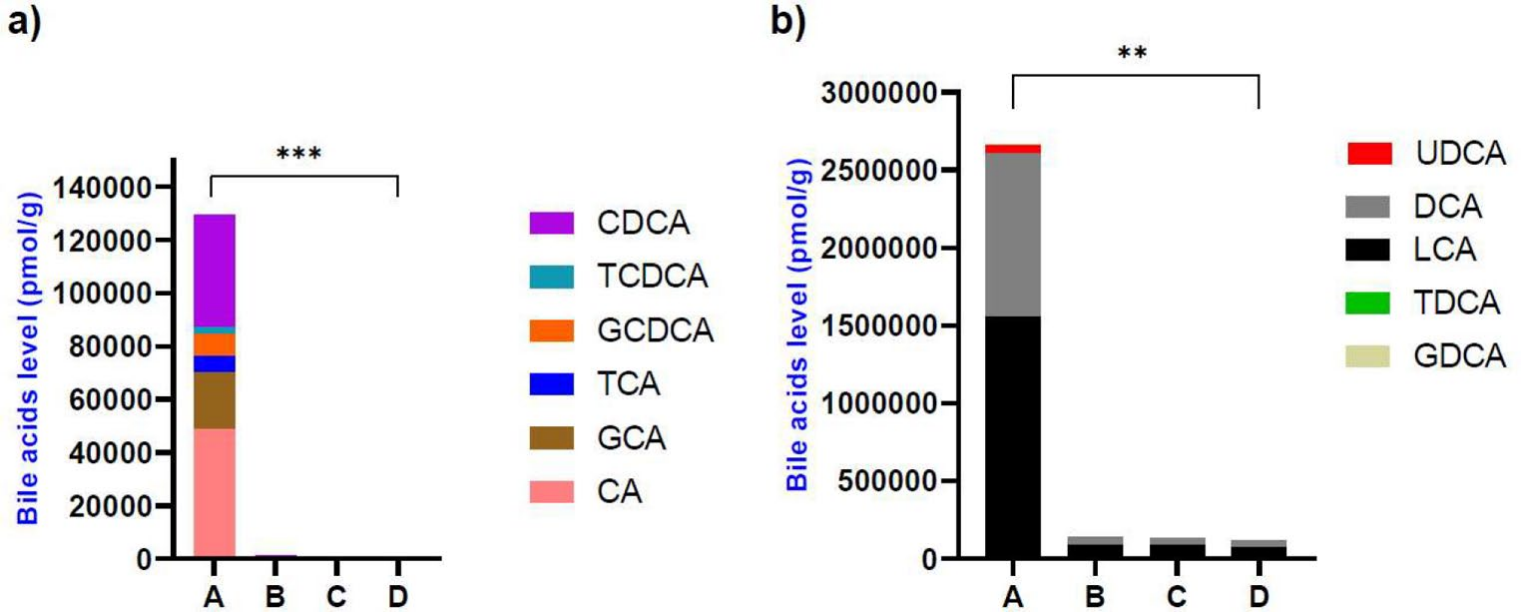
Measurement of bile acid levels in four stool samples from donors: **A**, unprocessed stool; **B**, stool suspension without glycerol, **C**; stool suspension with glycerol 10% and **D**; lyophilized stool suspension. (**a**) Primary bile acids, (**b**) Secondary bile acids. LCA, Lithocholic acid; DCA, Deoxycholic acid; UDCA, Ursodeoxycholic acid; TDCA, Taurodeoxycholic acid; GDCA, Glycodeoxycholic acid; CA, Cholic acid; GCA, Glycocholic acid; TCA, Taurocholic acid; GCDCA, Glycochenodeoxycholic acid; TCDCA, Taurochenodeoxycholic acid; CDCA, Chenodeoxycholic acid. Bile acid concentrations are reported in pmol/g

We observed a notable difference in the total bile acid concentrations among various FM preparations (Friedman test, *p* < 0.0001). Post hoc analysis revealed that the levels were significantly higher in the unprocessed stool (254,472 ± 531,042 pmol/g) compared to both the lyophilized samples (10,601 ± 24,191 pmol/g; *p* < 0.0001) and the processed stool suspension with glycerol (12,394 ± 28,266 pmol/g; *p* = 0.005). Furthermore, in the processed stool suspension without glycerol, the total bile acids were elevated at 12,673 ± 29,026 pmol/g compared to the lyophilized samples (*p* = 0.005). Regarding the primary bile acids, we found a significant difference in their total concentrations across different preparations (Friedman test, *p* < 0.0001). Dunn’s post-hoc analysis revealed higher levels in the unprocessed stool (21562 ± 19687 pmol/g) compared to the lyophilized samples (106.8 ± 139.3 pmol/g; *p* = 0.0003), while compared to the suspensions preparations (with glycerol: 151.4 ± 222.3 pmol/g, *p* = 0.083; without glycerol: 167.3 ± 217.2 pmol/g, *p* = 0.705) no significant differences were observed (Fig. 4).

Furthermore, secondary bile acids were also significantly different across different preparations (Friedman test, *p* < 0.0001), with higher levels in unprocessed stool (533,963 ± 724,925 pmol/g) than in lyophilized samples (23,195 ± 33,159 pmol/g; *p* = 0.001), while there were no significant differences between the other preparations (with glycerol: 27,086 ± 38,765 pmol/g, *p* = 0.164; without glycerol: 27,679 ± 39,877 pmol/g, *p* = 0.849) (Fig. 4). Lithocholic acid (LCA) and deoxycholic acid (DCA) were the most prevalent bile acids determined (Table S10), where their levels were significantly higher in the unprocessed stool (LCA: 1,559,424 ± 609,149 pmol/g; DCA: 1,045,361 ± 427,066 pmol/g) than in lyophilized samples (LCA: 72,233 ± 29,019 pmol/g, *p =* 0.037; DCA: 43,246 ± 17,939 pmol/g, *p =* 0.037), with no significant differences in their concentrations between the other FM preparations.

### Results of 16S rRNA gene amplicon sequencing

**Fig. 5:**
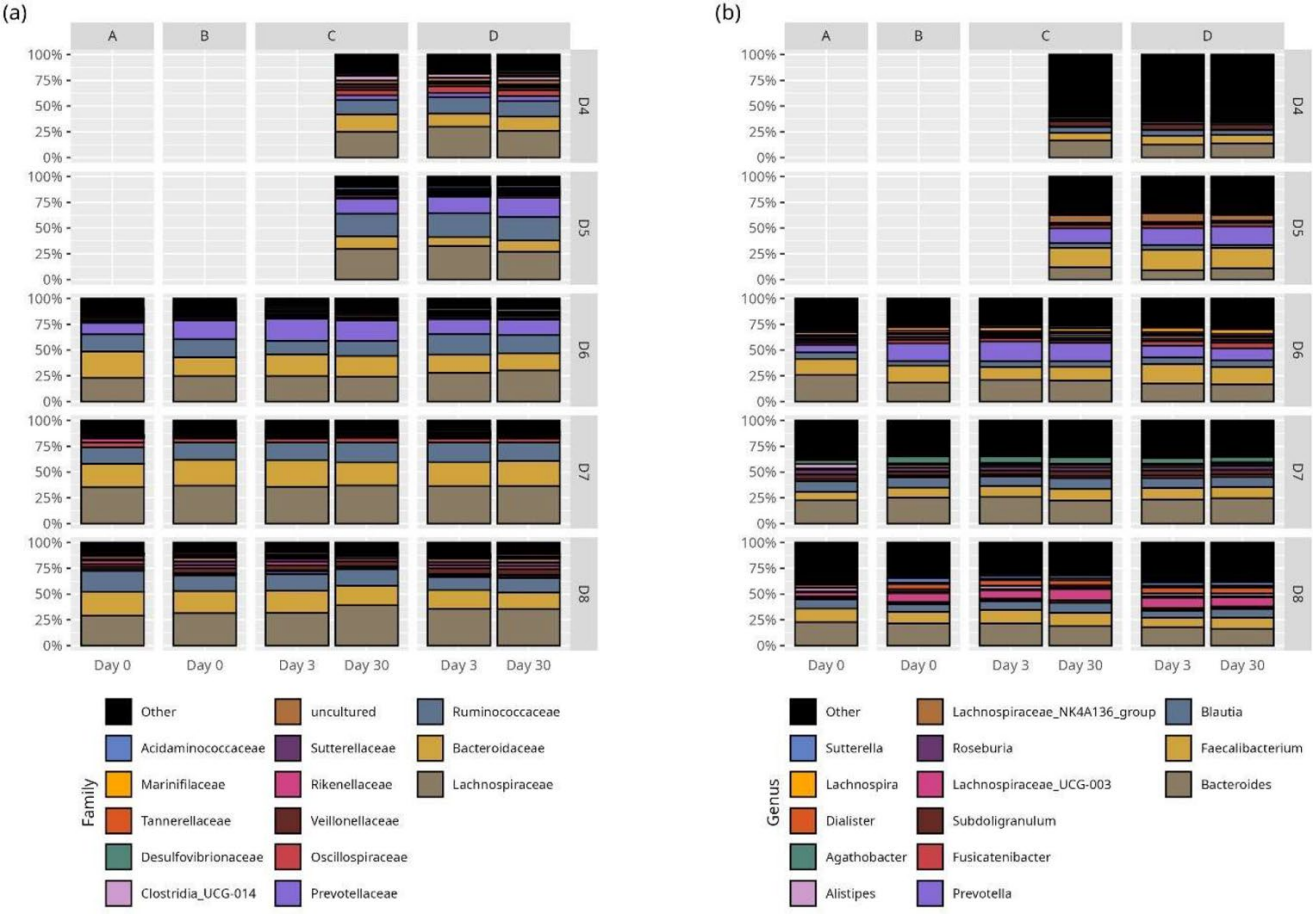
Effect of stool processing, freezing, and lyophilization on microbiota composition. Taxa bar plot representing the relative abundance of the fourteen most abundant bacterial taxa on family (**a**) and genus (**b**) level. No significant difference for any taxon depending on different preparations and storage conditions was observed. A; fresh unprocessed stool, B; fresh stool suspension, C; frozen stool suspension with glycerol, D; lyophilized stool suspension. D4, D5, D6, D7, D8 stands for different stool donors. Note: The unprocessed, processed, and frozen Day 3 samples for donors D4 and D5 were lost during laboratory processing

The taxonomic compositions of all preparations (fresh unprocessed stool, fresh processed stool suspension, frozen stool suspension and lyophilized stool suspension) are shown in Fig. 5. The taxonomic compositions of the donor samples in the pilot study are shown in the supplementary material, Fig. S3.

**Fig. 6:**
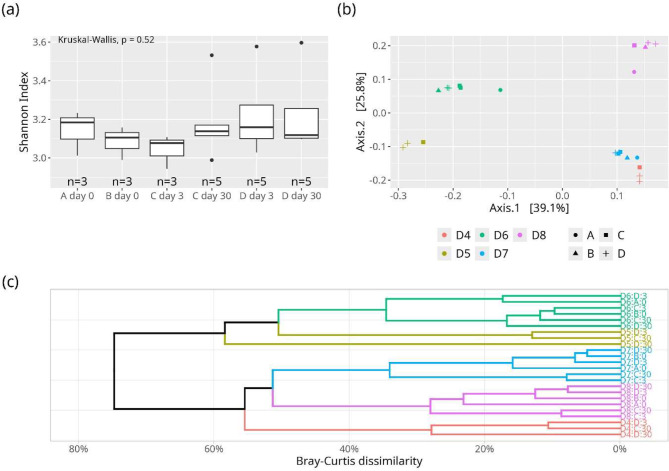
Comparison of the alpha and beta diversity of the different fecal microbiota preparations. (**a**) The different preparations and storage times did not affect the alpha diversity (i.e. the Shannon Index; Kurskal-Wallis *p* = 0.52). (**b**, **c**) Beta-diversity (Bray Curtis distance) visualized by principal component analysis (PCoA) (**b**) and hierarchical clustering dendrogram (**c**). Permutational multivariate analysis of variance using distance matrices (PERMANOVA) indicates that the majority of the observed dissimilarity between the samples is attributed to the different donor IDs (r2 = 0.92, *p* = 0.001). A; fresh unprocessed stool, B; fresh stool suspension, C; frozen stool suspension with glycerol, D; lyophilized stool suspension

Freezing and lyophilization did not affect the alpha-diversity of the donor samples (e.g., Shannon index in frozen stool suspension on day 30 vs. lyophilized stool suspension on day 30: 3.18 ± 0.2 vs. 3.23 ± 0.2, *p* = 0.52; Fig. 6a and Table S11).

Beta-diversity analyses, in this case the Bray-Curtis Dissimilarity, indicated that the majority of the observed dissimilarity between the samples was attributable to the different donor IDs (Fig. 6b; PERMANOVA: r^2^ = 0.92, F = 71.9, *p* = 0.001). Importantly, this analysis highlights that preparations from the same donor clustered, irrespective of whether they were fresh, frozen, or lyophilized (PERMANOVA: r^2^ = 0.02, F = 2.6, *p* = 0.011). Additionally, the composition of the samples remained unaffected by the storage duration (PERMANOVA: r^2^ = 0.002, F = 0.56, *p* = 0.688, Fig. 6b and c).

These findings were further validated through analysis of similarities (ANOSIM). The results from the ANOSIM analysis also corroborated these findings, highlighting that donor ID was the only significant factor for different samples clustering (*R* = 1, *p* = 0.001). Conversely, factors such as the preparation (*R* = -0.118, *p* = 1) or storage duration (*R* = -0.055, *p* = 0.98) did not exert a significantly impact the composition of the samples. The results do not change significantly using the generalized UniFrac distance (Supplementary material, Fig. S4).

### Preparation of LFM capsules

**Fig. 7:**
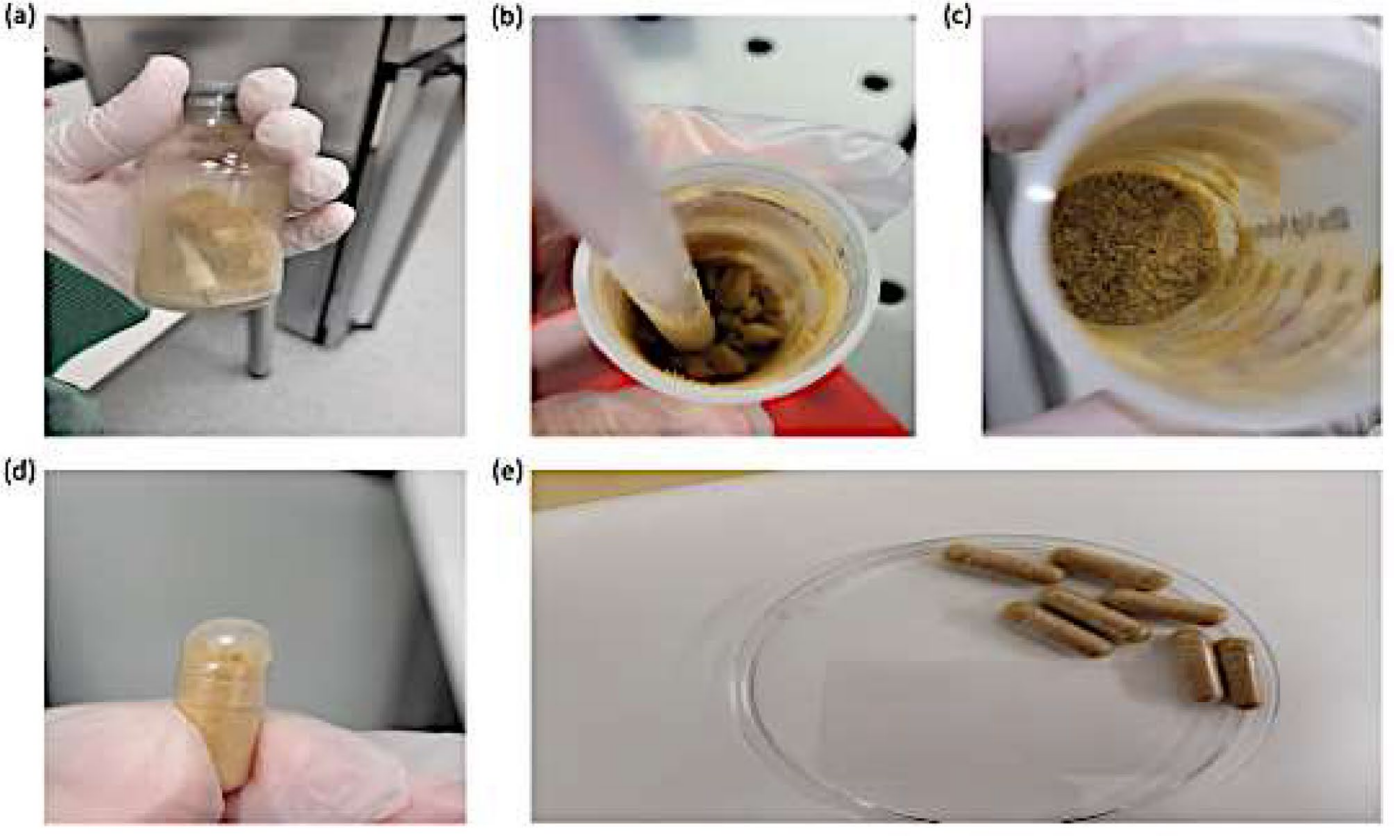
Photos of lyophilized fecal microbiota (LFM). (**a**) LFM cake after lyophilization (**b**, **c**) grinding of the LFM cake into powder form using a spatula (**d**, **e**) Encapsulation of LFM into size 0 delayed-release hydroxpropyl methylcellulose capsules (DRcaps^®^, Capsugel)

The average yield of lyophilized powder per standard stool donation (50 g) was 2.48 g ± 0.38 g lyophilized cake that was ground into a powder by use of a spatula (maximum was 3 g, minimum was 1.84 g). Delayed-release hydroxpropyl methylcellulose capsules size 0 (DRcaps^®^, Capsugel, Lonza, Basel, Switzerland) were manually filled each with 0.2 g of the lyophilized powder (Fig. 7). The mean recovered viable bacterial cell concentration in the LFM product of 5 stool donations on day 3 was 4.62 × 10^10^ cells/g of initial donor stool and 8.05 × 10^10^ total cells/g, respectively. Hence, a standard stool donation (50 g) would result in 2.31 × 10^12^ viable cells and 4.02 × 10^12^ total bacterial cells contained in 2.48 ± 0.38 g lyophilized product. Given that each capsule may hold 0.2 g of powder, one donation would result in 12.4 ± 2 capsules (10.5–14 capsules); each containing about 1.86 × 10^11^ viable bacterial cells and 3.24 × 10^11^ total bacterial cells.

### Residual water measurement

The residual moisture levels varied greatly among the donations, but also within the donations themselves. Water content post primary drying was nearly two times higher than after secondary drying (Supplementary material, Table S12 and Fig. S5). Further, the measured water contents after secondary drying were in a very narrow range and did not show a high deviation from the median compared to the measured water contents after primary drying. The mean residual moisture content was significantly higher in samples after primary drying than after secondary drying (14% vs. 7%, respectively) (unpaired t-test *r* = 0.649, *p* = 0.001).

## Discussion

Frozen oral FM capsules are well established for the treatment of rCDI. Their practical application is, however, constrained by difficulties in terms of aesthetics, storage, transportation, and handling. To address these limitations, the concept of lyophilization emerged as a solution. As the process of lyophilization can impair bacterial viability by impacting membrane integrity (Wagman 1960; Jiang et al. 2018), we developed a comprehensive in vitro validation approach.

To assess the bacterial viability in different FM products, we utilized both the QUANTOM Tx™ microbial cell counter and conventional culturing techniques. Based on the cell counter results, we observed a 6-fold drop in total cells and a 5.77-fold drop in viable cells between unprocessed and freshly processed stool on day 0. As this loss was not restricted to anaerobic bacteria, we hypothesize that the mechanical effects of filtration and centrifugation may have contributed to these changes. Alternatively, a considerable number of cells might have been lost due to inefficient separation during centrifugation. Unfortunately, we did not measure viable cells in the initial supernatant, such that this issue will need to be clarified through future analyses.

The clinical relevance of these findings remains unclear, as the majority of clinical trials have successfully treated rCDI using stool processed in this same manner (Cammarota et al. 2017; Mullish et al. 2018; Minkoff et al. 2023). In the present analysis, there is a non-significant reduction in the viable cell concentration between the lyophilized stool suspension and the frozen suspension after one month of storage. The percentage of viable cells after one month of storage at -80 °C was 69% in the frozen suspension and 57% in the lyophilized stool suspension stored at 4 °C. It dropped to 47% after three months of storage (Table S8). Our findings were in line with those of Staley et al., who examined membrane integrity using the BacLight live/dead membrane integrity assay throughout a two-month storage period and found 56% membrane integrity (Staley et al. 2017).

Our findings with respect to anaerobic and aerobic recovery of CFU/g showed a drop between the unprocessed stool sample and the stool suspension, as well as between the frozen and lyophilized preparations on day 3 and 30. These drops were more pronounced in anaerobic, than in aerobic bacteria, as previously reported (Jiang et al. 2018) and explained by exposure of the fecal samples to oxygen during processing (Papanicolas et al. 2019). The observation that culture independent assessment of viable bacterial cell counts did not differ significantly between fresh, frozen and lyophilized preparations (Fig. 2b) supports the hypothesis that the limited cultural recovery of anaerobes may be attributed to our culture approach, rather than an actual drop in anaerobic bacteria in the processed samples. In this context, the duration of incubation we selected may have played an important role. Our aerobic and anaerobic cultures were incubated for 24 and 48 h, respectively, as previously published by Bénard et al. (Bénard et al. 2023). Other studies, however, favored longer incubation periods (Reygner et al. 2020; Bilinski et al. 2022).

In a second step, we sought to ascertain whether secondary drying has a detrimental effect on bacterial viability. Although there was no significant difference in viable cell counts before and after secondary drying, one might postulate a slight tendency towards a higher viability after secondary drying (paired t-test, *p* = 0.331) (Supplementary material, Table S9 and Fig. S1). Possible reasons include a lack of declustering by the QUANTOM Tx™ cell counter, improper sample preparation or insufficient resuspension of the lyophilized samples. The higher viable cell concentration after secondary drying may also have resulted from the decreased water content. Thus, we can conclude, that secondary drying did not have a negative impact on the viability of bacterial cells.

One of the proposed mechanisms of action of FMT in rCDI relies on the regulatory activity of primary and secondary bile acids. Primary bile acids promote *C. difficile* germination, while secondary bile acids inhibit vegetative growth and toxin production (Sorg and Sonenshein 2008, 2010; Khoruts and Sadowsky 2016). Farowski et al. observed that the presence of the secondary bile acid lithocholic acid (LCA) within recipient stool samples is a good predictor of FMT success, with its concentration being comparable to that in donor samples (Farowski et al. 2019). In our study, the most abundant bile acids in the different FM aliquots were LCA and DCA. In a previous analysis on the detection of primary and secondary bile acids in lyophilized stool samples, concentrations were not found to be altered significantly after lyophilization (Hu et al. 2023). This finding contrasts with our results, as the concentration of primary and secondary bile acids was significantly reduced in the lyophilized samples. While we filtered and centrifuged the baseline fecal samples prior to lyophilization, Hu et al. performed the lyophilization directly without any further manipulation of the samples. We therefore hypothesize that our stool processing steps may have contributed to the decline of bile acid concentrations in the lyophilized samples. The difference between the loss of primary and secondary bile acids may be explained by a higher solubility of primary bile acids in the supernatant that is discarded after centrifugation. This hypothesis will need to be validated in follow-up analyses.

The study evaluated the impact of lyophilization on gut microbiota diversity using next-generation sequencing. Results showed no effect on alpha diversity indices after 1 month of storage at 4 °C. No significant changes in intra-donor microbiome composition across different FMT preparations were observed. Since this approach does not distinguish viable from non-viable bacteria, we suggest a follow-up study that combines next-generation sequencing with a pretreatment of samples with propidium monoazide (PMA) dye that prevents amplification of dead cells (Nocker et al. 2007).

One of the most important advantages of lyophilization is the reduction in the number of required capsules due to water sublimation. This was confirmed by our analysis, as we could reduce the number of required capsules from 30 to 10.5–14 capsules size 0, which is within the range documented in other studies (Jiang et al. 2018; Reigadas et al. 2020).

The residual moisture content after primary drying was expected to be 5–20% and 1–5% after the secondary drying (PharmTech 2018; Meirowitz 2019). In our study, the residual moisture content after primary drying was within the expected range (14%), the residual moisture content for the secondary dried samples deviated from this in their arithmetic mean (7%) (Pearson’s *r* = 0.893, *p* = 0.003), (Supplementary material, Table S12 and Fig. S5). One possible source of error was the reliance on visual observation to determine the color change. For this project, volumetric Karl-Fischer titration was selected due to its cost-effectiveness and ease of use. The endpoint, indicating that all water had reacted, was marked by a color change to yellowish-brown, when iodine was no longer reduced to iodide. Since the stool sample suspension with the added Solvent E already had a brown colour from the beginning, it was difficult to recognise the colour change, and it is possible that the colour change was only recognised by the operator’s eye when the colouring became more intense. To obtain more precise results, a titration with an alternative method should be carried out, e.g. a coulometric Karl Fischer titration.

The key strengths of our study were the multiple techniques used to validate the viability and diversity of the LFM after 1 and 3 months of storage, as well as the assessment of the primary and secondary bile acids. Furthermore, we investigated the viability of the raw stool prior to processing and discovered that the majority of the decline in viability and bile acids happened primarily during processing and not during freezing or lyophilization. Based on the determined concentration of viable cells, we conclude that the secondary drying step can be integrated into the process for lyophilization of donor stool suspension for FMT. Our study has some limitations, such as the small sample size of donors (*n* = 5) that does not allow for generalizability of our findings to a broader range of individual donations. The incubation period for culturing aerobes was limited to 24 h and that of anaerobes to 48 h, and our viability study was limited to a maximum of only 3 months. Likewise, we did not assess the viability of the LFM capsules. To address these constraints, studies with a larger sample size and longer follow-up periods of up to 1–2 years are necessary.

## Conclusion

We provide a comprehensive in vitro concept for the validation of lyophilized GMP-produced FM capsules, with a special focus on bacterial viability. The aim is to facilitate FMT access to patients by reducing the number of capsules, minimizing unpleasant odours, simplifying storage and delivery, and lowering prices. Our research indicates that the bacterial viability and bile acid composition of LFM, stored at 4 °C (with 5% trehalose), remained stable without significant loss when compared to freshly processed stool suspension or frozen FM in 10% glycerol.

## Electronic supplementary material

Below is the link to the electronic supplementary material.

Supplementary Material 1

## Data Availability

The sequencing data generated and analysed in this study is available in the NCBI Sequence Read Archive under the BioProject accession number PRJNA1208555. Further inquiries can be directed to the corresponding author.
