# Supplementary material for: In vitro validation concept for lyophilized fecal microbiota products with a focus on bacterial viability
Source: World J Microbiol Biotechnol. 2025 Feb 27;41(3):83. doi: 10.1007/s11274-025-04291-0 (PMC11865215; doi:10.1007/s11274-025-04291-0)

Supporting Information for

**In vitro validation concept for lyophilized fecal microbiota products with a focus on bacterial viability**

Sara A. Sedeek^1, 2^, [Fedja Farowski](https://pubmed.ncbi.nlm.nih.gov/?term=Farowski+F&cauthor_id=36651357)^1, 3, 4^, Stella Youssafi^3^, Anastasia Tsakmaklis^3, 4^, Susanne Brodesser^5^, Madiha M. El-Attar^2^, Mohamed Omar Abdelmalek^2^, and Maria J.G.T. Vehreschild^1,3,4^

1- Goethe University Frankfurt, University Hospital Frankfurt, Department of Internal Medicine II, Infectious Diseases, Frankfurt am Main, Germany

2- Department of Tropical Medicine and Gastroenterology, Assiut University, Assiut, Egypt

3- University of Cologne, Faculty of Medicine and University Hospital Cologne, Department I of Internal Medicine, Centre for Integrated Oncology Aachen Bonn Cologne Duesseldorf, Germany

4- German Centre for Infection Research (DZIF), Partner Site Bonn-Cologne, Germany

5- University of Cologne, Faculty of Medicine and University Hospital of Cologne, Cluster of Excellence Cellular Stress Responses in Aging-associated Diseases (CECAD), Cologne, Germany

Corresponding author: Maria J.G.T. Vehreschild, Email Address: vehreschild@med.uni-frankfurt.de

**Table S1**

Baseline characteristics of the stool donors

| **Donor no.** | **Sex** | **Age**** |
| --- | --- | --- |
| *Donor 1 | male | 31 |
| *Donor 2 | male | 45 |
| *Donor 3 | female | 44 |
| Donor 4 | female | 37 |
| Donor 5 | male | 33 |
| Donor 6 | female | 41 |
| Donor 7 | female | 57 |
| Donor 8 | male | 31 |

*The data of the first 3 donors were excluded from the final results. ** median age is 39 years.

**Table S2**

Measurement of the total bacterial cell concentration (cells/g) in different microbiota preparations of 8 stool donors using the QUANTOM Tx™ microbial cell counter (donors 1-3=pilot study, donors 4-8=final model)

| **Donor no.** | **Fresh unprocessed stool** | **Fresh processed suspension** | **Frozen suspension, day 3** | **Frozen suspension, day 30** | **Lyophilized stool suspension, day 3** | **Lyophilized stool suspension, day 30** | **Lyophilized stool suspension day 90**** |
| --- | --- | --- | --- | --- | --- | --- | --- |
| *Donor 1 | 4.74E+09 | 5.74E+04 | 8.05E+10 | 7.36E+10 | 2.31E+10 | 3.08E+10 | Not done |
| *Donor 2 | 3.82E+11 | 7.05E+10 | 1.11E+11 | 6.83E+10 | 3.45E+10 | 6.22E+10 |  |
| *Donor 3 | 5.93E+11 | 9.78E+10 | 5.59E+10 | 6.41E+10 | 3.52E+10 | 2.91E+10 |  |
| Donor 4 | 5.26E+11 | 1.06E+11 | 1.20E+11 | 1.57E+11 | 9.05E+10 | 1.10E+11 | 1.64E+11 |
| Donor 5 | 4.78E+11 | 9.74E+10 | 2.10E+11 | 1.25E+11 | 9.37E+10 | 1.02E+11 | 1.15E+11 |
| Donor 6 | 3.86E+11 | 6.85E+10 | 3.96E+10 | 5.65E+10 | 7.28E+10 | 5.65E+10 | 7.64E+10 |
| Donor 7 | 6.32E+11 | 7.25E+10 | 7.94E+10 | 1.04E+11 | 6.78E+10 | 6.55E+10 | 6.97E+10 |
| Donor 8 | 4.86E+11 | 6.71E+10 | 7.79E+10 | 6.38E+10 | 7.75E+10 | 5.68E+10 | 8.55E+10 |

*The data of the first 3 donors were excluded from the final results.

** It was done only for the last 5 donations

**Table S3**

Measurement of the viable bacterial cell concentration (cells/g) in different microbiota preparations of 8 stool donors using the QUANTOM Tx™ microbial cell counter (donors 1-3=pilot study, donors 4-8=final model)

| **Donor no.** | **Fresh unprocessed stool** | **Fresh processed suspension** | **Frozen suspension, day 3** | **Frozen suspension, day 30** | **Lyophilized stool suspension, day 3** | **Lyophilized stool suspension, day 30** | **Lyophilized stool suspension, day 90**** |
| --- | --- | --- | --- | --- | --- | --- | --- |
| *Donor 1 | 2.00E+09 | 6.16E+08 | 8.79E+10 | 3.58E+10 | 4.59E+09 | 9.76E+09 | Not done |
| *Donor 2 | 3.04E+11 | 5.43E+10 | 1.03E+11 | 5.83E+10 | 2.13E+10 | 4.35E+10 |  |
| *Donor 3 | 4.05E+11 | 7.21E+10 | 4.81E+10 | 7.22E+10 | 1.63E+10 | 1.05E+10 |  |
| Donor 4 | 6.19E+11 | 7.24E+10 | 1.07E+11 | 8.87E+10 | 5.33E+10 | 3.17E+10 | 5.41E+10 |
| Donor 5 | 3.04E+11 | 9.13E+10 | 1.77E+11 | 1.05E+11 | 6.53E+10 | 7.46E+10 | 6.12E+10 |
| Donor 6 | 2.12E+11 | 3.81E+10 | 4.17E+10 | 4.48E+10 | 4.40E+10 | 4.57E+10 | 3.96E+10 |
| Donor 7 | 3.48E+11 | 5.93E+10 | 6.75E+10 | 7.28E+10 | 3.45E+10 | 3.31E+10 | 4.08E+10 |
| Donor 8 | 2.68E+11 | 4.25E+10 | 3.27E+10 | 4.02E+10 | 3.42E+10 | 3.88E+10 | 4.37E+10 |

*The results of the first 3 donors were excluded from the final analysis.

** It was done only for the last 5 donations

**Table S4**

Culture of anaerobes (CFU/g) of 8 stool donors in different preparations (donors 1-3=pilot study, donors 4-8=final model)

| Donor no. | Fresh unprocessed stool | Fresh processed suspension | Frozen suspension, day 3 | Frozen suspension, day 30 | Lyophilized stool suspension, day 3 | Lyophilized stool suspension, day 30 |
| --- | --- | --- | --- | --- | --- | --- |
| *Donor 1 | 1.02E+07 | 4.36E+06 | 1.89E+06 | 8.04E+07 | 6.37E+06 | 2.39E+05 |
| *Donor 2 | 1.01E+08 | 6.51E+07 | 8.09E+07 | 1.26E+08 | 1.66E+07 | 2.37E+06 |
| *Donor 3 | 3.88E+08 | 1.07E+08 | 9.54E+07 | 6.23E+07 | 2.65E+06 | 9.15E+04 |
| Donor 4 | 4.87E+08 | 1.04E+08 | 1.89E+08 | 9.68E+07 | 3.47E+07 | 4.62E+07 |
| Donor 5 | 6.08E+08 | 1.26E+08 | 1.18E+08 | 1.08E+08 | 3.14E+07 | 3.99E+07 |
| Donor 6 | 4.66E+08 | 9.13E+07 | 9.19E+07 | 9.96E+07 | 5.62E+07 | 5.72E+07 |
| Donor 7 | 5.68E+08 | 1.13E+08 | 1.38E+08 | 1.43E+08 | 2.32E+07 | 8.79E+06 |
| Donor 8 | 5.59E+08 | 8.75E+07 | 9.39E+07 | 1.00E+08 | 1.13E+07 | 2.69E+07 |

*The results of the first 3 donors were excluded from the final analysis.

**Table S5**

Culture of aerobes (CFU/g) of 8 stool donors in different preparations (donors 1-3=pilot study, donors 4-8=final model)

| Donor no. | Fresh unprocessed stool | Fresh processed suspension | Frozen suspension, day 3 | Frozen suspension, day 30 | Lyophilized stool suspension, day 3 | Lyophilized stool suspension, day 30 |
| --- | --- | --- | --- | --- | --- | --- |
| *Donor 1 | 5.62E+04 | 2.89E+05 | 7.37E+05 | 6.97E+05 | 1.07E+05 | 5.47E+04 |
| *Donor 2 | 6.10E+04 | 1.39E+06 | 1.35E+06 | 1.13E+06 | 8.71E+03 | 2.03E+04 |
| *Donor 3 | 1.18E+07 | 7.21E+05 | 6.78E+05 | 4.49E+05 | 2.81E+04 | 5.32E+03 |
| Donor 4 | 7.96E+07 | 5.35E+06 | 5.01E+06 | 6.17E+06 | 3.34E+04 | 6.29E+04 |
| Donor 5 | 2.77E+07 | 1.74E+06 | 1.77E+06 | 1.87E+06 | 3.35E+05 | 8.28E+05 |
| Donor 6 | 1.15E+06 | 3.28E+05 | 4.22E+05 | 5.10E+05 | 1.11E+05 | 1.11E+05 |
| Donor 7 | 9.49E+05 | 9.27E+04 | 2.39E+05 | 1.39E+05 | 8.23E+03 | 7.26E+03 |
| Donor 8 | 1.03E+07 | 7.75E+05 | 4.17E+04 | 6.32E+05 | 1.77E+05 | 2.10E+05 |

*The results of the first 3 donors were excluded from the final analysis.

**Table S6**

Culture of spores (CFU/g) of 8 stool donors in different preparations (donors 1-3=pilot study, donors 4-8=final model)

| **Donor no.** | **Fresh unprocessed stool** | **Fresh processed suspension** | **Frozen suspension, day 3** | **Frozen suspension, day 30** | **Lyophilized stool suspension, day 3** | **Lyophilized stool suspension, day 30** |
| --- | --- | --- | --- | --- | --- | --- |
| *Donor 1 | 0 | 1.48E+03 | 2.14E+03 | 2.47E+03 | 3.56E+04 | 2.03E+03 |
| *Donor 2 | 4.07E+03 | 3.13E+04 | 4.40E+03 | 5.92E+04 | 1.21E+05 | 3.70E+03 |
| *Donor 3 | 2.03E+04 | 2.47E+03 | 5.76E+03 | 5.76E+03 | 5.52E+04 | 5.44E+02 |
| Donor 4 | 1.49E+06 | 1.07E+04 | 8.48E+04 | 4.60E+04 | 1.28E+05 | 1.51E+04 |
| Donor 5 | 1.84E+06 | 8.95E+05 | 9.76E+04 | 1.60E+05 | 8.76E+05 | 5.86E+04 |
| Donor 6 | 5.89E+03 | 1.15E+04 | 2.37E+04 | 1.74E+04 | 6.34E+04 | 2.18E+04 |
| Donor 7 | 4.55E+04 | 1.05E+05 | 2.42E+04 | 6.60E+05 | 1.35E+05 | 2.07E+04 |
| Donor 8 | 1.71E+05 | 3.75E+04 | 4.27E+04 | 1.54E+04 | 9.15E+04 | 1.15E+04 |

*The results of the first 3 donors were excluded from the final analysis.

**Table S7**

Arithmetic mean of the subsequent triple measurement of the total cell concentration (cells/g) across all time points (t_1_-t_5_) and donors (A-F). [t_1_: fresh processed stool suspension; t_2_: freezing in the ultra-low deep freezer; t_3_: freezing in the freeze-dryer; t_4_: primary drying; t_5_: secondary drying]

| Donor-ID | Total cell conc. in cells/g (t_1_) | Total cell conc. in cells/g (t_2_) | Total cell conc. in cells/g(t_3_) | Total cell conc. in cells/g (t_4_) | Total cell conc. in cells/g (t_5_) |
| --- | --- | --- | --- | --- | --- |
| A | 1.01E+11 | 6.99E+10 | 6.63E+10 | 8.41E+10 | 1.02E+11 |
| B | 4.32E+10 | 5.33E+10 | 5.39E+10 | 6.75E+10 | 6.36E+10 |
| C | 3.11E+10 | 7.39E+10 | 7.34E+10 | 9.69E+10 | 8.16E+10 |
| D | 1.02E+11 | 7.71E+10 | 7.17E+10 | 6.41E+10 | 6.15E+10 |
| E | 8.74E+10 | 1.14E+11 | 1.12E+11 | 7.81E+10 | 7.96E+10 |
| F | 1.16E+11 | 7.76E+10 | 1.36E+11 | 5.06E+10 | 7.67E+10 |

**Table S8**

Percent of viable cells in each fecal microbiota preparation in 5 stool donors based on QUANTOM Tx microbial cell counter (final model only):

| **Sample** | **Viable (cells/g)** | **Non-Viable (cells/g)** | **Total (cells/g)** | **Viable %** |
| --- | --- | --- | --- | --- |
| **Raw stool** | 3.50E+11 | 1.51E+11 | 5.02E+11 | **69.83%** |
| **Fresh suspension** | 6.07E+10 | 2.16E+10 | 8.23E+10 | **73.74%** |
| **Frozen, D3** | 8.52E+10 | 2.01E+10 | 1.05E+11 | **80.91%** |
| **Frozen, D30** | 7.03E+10 | 3.09E+10 | 1.01E+11 | **69.49%** |
| **Lyophilized, day 3** | 4.62E+10 | 3.42E+10 | 8.05E+10 | **57.45%** |
| **Lyophilized, day 30** | 4.48E+10 | 3.35E+10 | 7.83E+10 | **57.23%** |
| **Lyophilized, day 90** | 4.79E+10 | 5.43E+10 | 1.02E+11 | **46.83%** |

**Table S9**

Arithmetic mean of the subsequent triple determination of the viable cell concentration (cells/g) across all time points t1-t5 and all six donors (A-F). [t_1_: fresh processed stool suspension; t_2_: freezing in the ultra-low deep freezer; t_3_: freezing in the freeze-dryer; t_4_: primary drying; t_5_: secondary drying]

| **Donor-ID** | **Viable conc. in cells/g (t_1_)** | **Viable cell conc. in cells/g (t_2_)** | **Viable cell conc. in cells/g (t_3_)** | **Viable cell conc. in cells/g (T_4_)** | **Viable cell conc. in cells/g (t_5_)** |
| --- | --- | --- | --- | --- | --- |
| **A** | 6.79E+10 | 8.55E+10 | 6.35E+10 | 5.03E+10 | 4.92E+10 |
| **B** | 2.69E+10 | 2.56E+10 | 1.97E+10 | 3.24E+10 | 3.44E+10 |
| **C** | 1.93E+10 | 3.64E+10 | 2.36E+10 | 3.68E+10 | 3.78E+10 |
| **D** | 4.60E+10 | 6.30E+10 | 5.09E+10 | 4.14E+10 | 3.86E+10 |
| **E** | 5.25E+10 | 4.57E+10 | 4.55E+10 | 3.14E+10 | 4.10E+10 |
| **F** | 8.48E+10 | 6.68E+10 | 7.40E+10 | 5.32E+10 | 5.55E+10 |

**Table S10**

Results of primary and secondary bile acids extraction (pmol /g) in the aliquots of four stool donors (final model only)

| **Bile acid**  **(mean ± SD, pmol /g)** | **Unprocessed stool** | **Stool suspension without glycerol** | **Stool suspension with glycerol** | **Lyophilized stool** **suspension** | ***p* value*** |
| --- | --- | --- | --- | --- | --- |
| 1-Cholic acid | 48570 ± 66142 | 357.9 ± 345.9 | 270.4 ± 242.5 | 248 ± 198.9 | 0.019 |
| 2-Glychochenodeoyxcholic  acid | 8710 ± 8778 | 57.69 ± 48.6 | 37.04 ± 28.1 | 35 ± 24.46 | 0.0009 |
| 3-Taurochenodeoxycholic acid | 2409 ± 2547 | 8.6 ± 9.07 | 5.56 ± 5.39 | 5.35 ± 4.26 | 0.0329 |
| 4-Chenodeoxycholic acid | 42231± 40824 | 519 ± 324.1 | 555.8 ± 607.9 | 319.2 ± 158.5 | 0.0417 |
| 5-Glycocholic acid | 21678 ± 29600 | 53.98 ± 45.75 | 35.49 ± 25.04 | 29.41 ± 19.57 | 0.0538 |
| 6-Taurocholic acid | 5775 ± 7525 | 6.88 ± 5.47 | 4.18 ± 2.07 | 3.93 ± 1.28 | 0.147 |
| 7-Ursodeoxycholic acid  (median) | 54644 ± 90009  (11838) | 589.5± 892.5  (152.6) | 471.9 ± 734.2  (125.7) | 440.6 ± 545.8  (192.3) | 0.019 |
| 8-Taurodeoxycholic acid | 1723 ± 1309 | 4.656 ± 4.704 | 3.441 ± 2.756 | 2.589 ± 1.710 | 0.031 |
| 9-Glycodeoxycholic acid | 8665 ± 5510 | 92.51 ± 67.15 | 55.06 ± 34.06 | 51.96 ± 38.86 | 0.0009 |
| 10- Lithocholic acid | 1559424 ± 609149 | 87685 ± 35012 | 84478 ± 44329 | 72233 ± 29019 | 0.0190 |
| 11-Deoxycholic acid | 1045361 ± 427066 | 50022 ± 27482 | 50421 ± 32092 | 43246 ± 17939 | 0.0329 |
| 12-Total bile acid concentration (pmol/g) | 2799190559.9 | 139398344.4 | 136338336.8 | 116615326.6 | <0.0001 |

*Friedman test performed to assess differences across multiple related groups.

**Table S11**

Shannon index of the different stool aliquots

| **Samples** | **Day of sequencing** | **N** | **Mean ± SD** |
| --- | --- | --- | --- |
| Fresh unprocessed stool | Day 0 | 3 | 3.14 ± 0.12 |
| Fresh processed suspension | Day 0 | 3 | 3.08 ± 0.08 |
| Frozen suspension | Day 3 | 3 | 3.04 ± 0.08 |
| Frozen suspension | Day 30 | 5 | 3.18 ± 0.2 |
| Lyophilized stool suspension | Day 3 | 5 | 3.22 ± 0.21 |
| Lyophilized stool suspension | Day 30 | 5 | 3.23 ± 0.21 |

**Table S12**

Arithmetic mean of the triple measurements of the water content of all samples after the primary drying (t_4_) and the secondary drying (t_5_) in %.

| Donor-ID | Water content in % (t_4_) | Water content in % (t_5_) |
| --- | --- | --- |
| A | **13.14** | **6.42** |
| B | **13.95** | **7.09** |
| C | **22.07** | **9.31** |
| D | **11.77** | **6.30** |
| E | **13.62** | **6.08** |
| F | **11.34** | **7.36** |


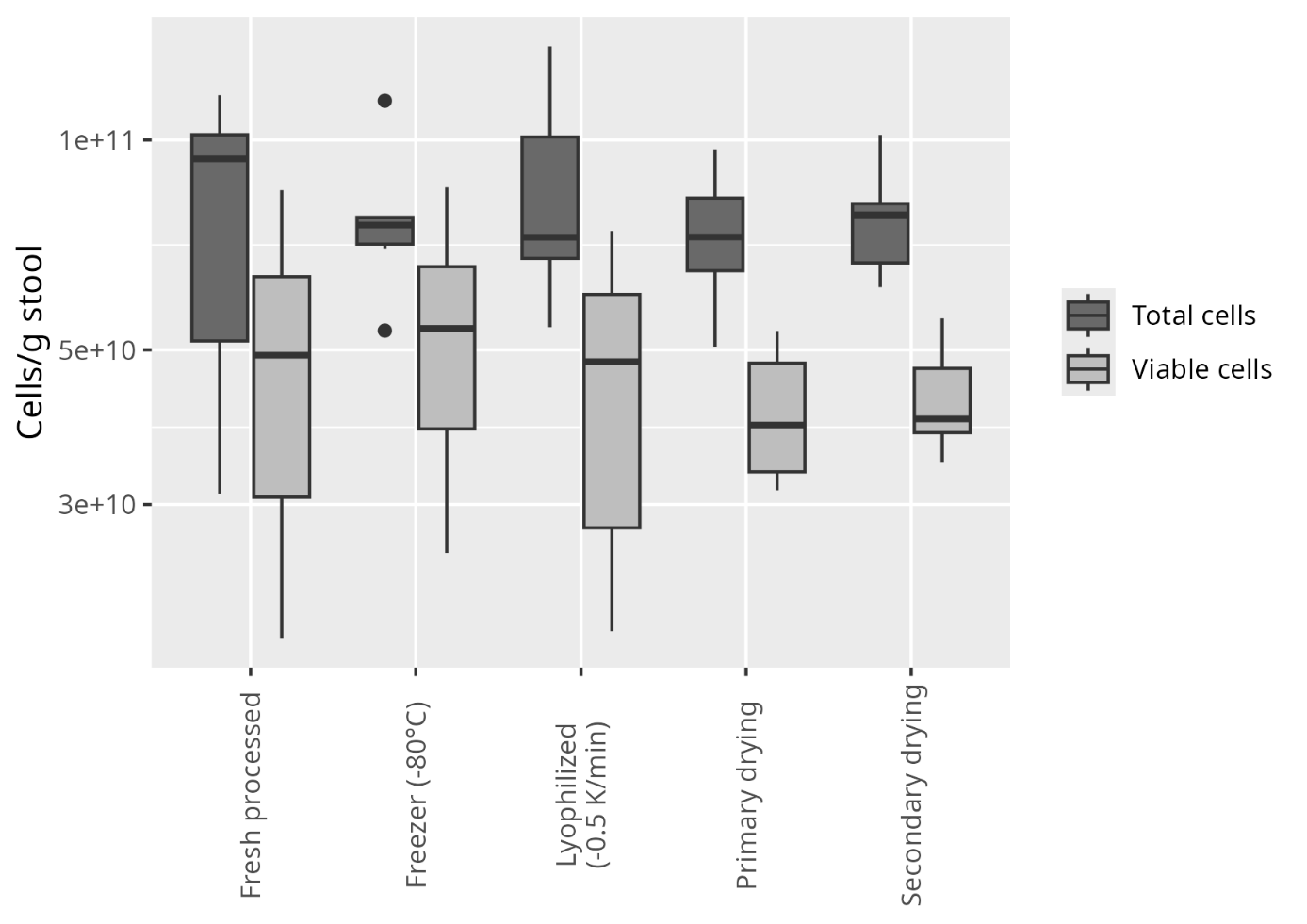


**Fig. S1** Boxplot of total and viable cell concentrations (cells/g raw stool) across different stages of the lyophilization process


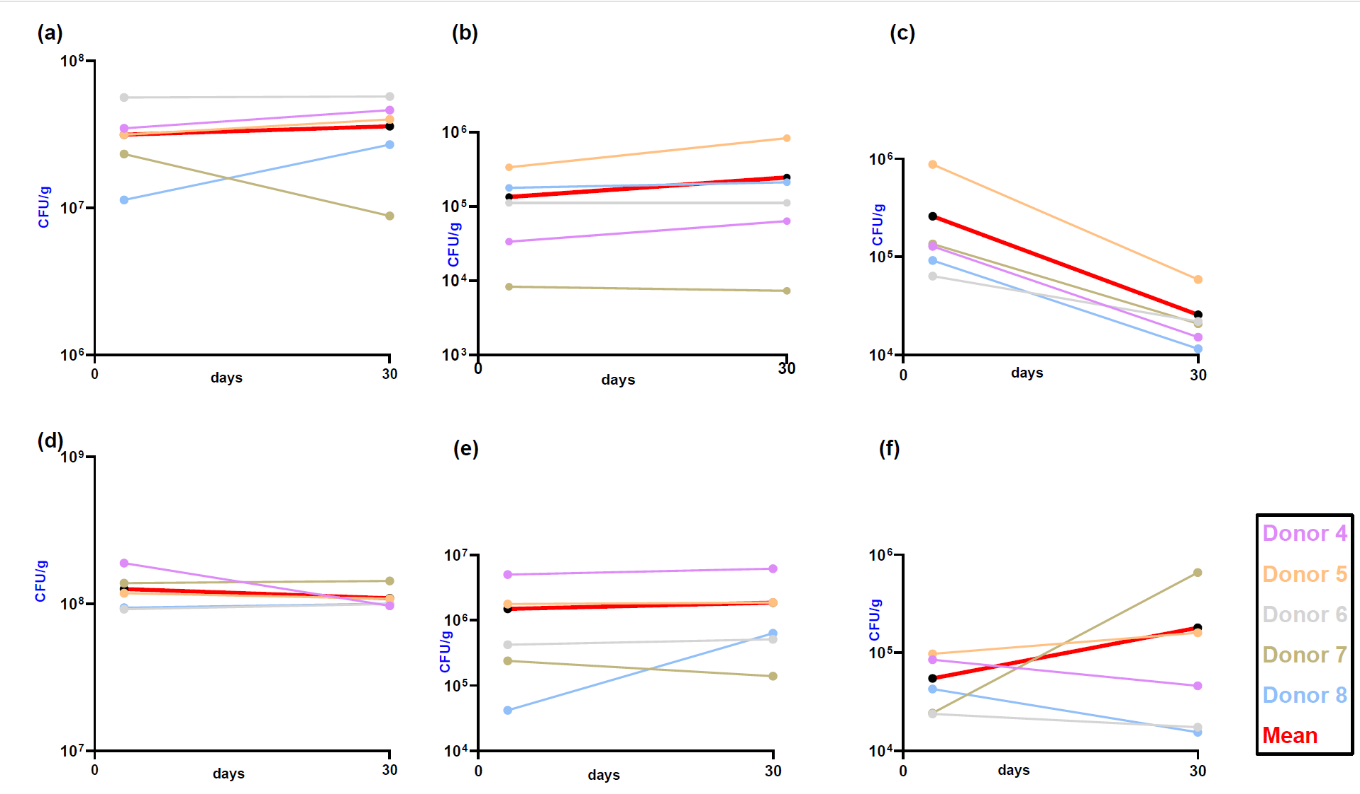


**Fig. S2** Spaghetti-plots showing the results of bacterial culture (CFU/g) on day 3 versus day 30 of: (1) The lyophilized stool suspension culture for (a) Anaerobes (b) Aerobes (c) Spores; and (2) the frozen suspension culture for (d) Anaerobes (e) Aerobes (f) Spores. No statistically significant reduction of the CFU/g for anaerobes, aerobes and spores culture of either the frozen suspension or the lyophilized stool suspension on day 30 versus day 3


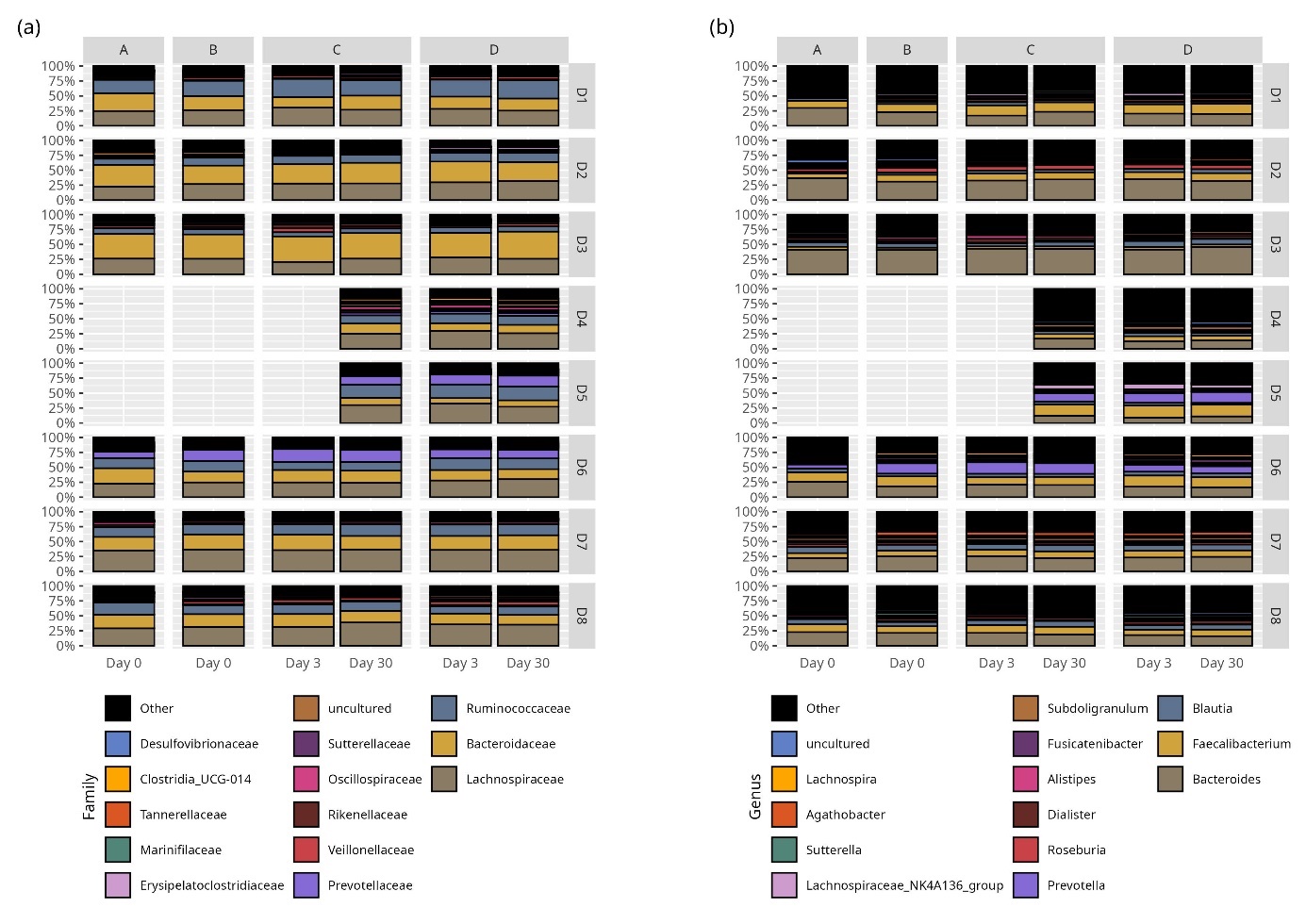


**Fig. S3** Effect of stool processing, freezing, and lyophilization on microbiota composition. Taxa bar plot representing the relative abundance of the fourteen most abundant bacterial taxa on family (a) and genus (b) level. No significant difference for any taxon depending on different and storage conditions preparations was observed. A; fresh unprocessed stool, B; fresh stool suspension, C; Frozen stool suspension with glycerol, D; lyophilized stool suspension. D1, D2, D3, D4, D5, D6, D7, D8 stands for different stool donors (donors 1-3=pilot study, donors 4-8=final model).
Note: The unprocessed, processed, and frozen Day 3 samples for donors D4 and D5 were lost during laboratory processing.


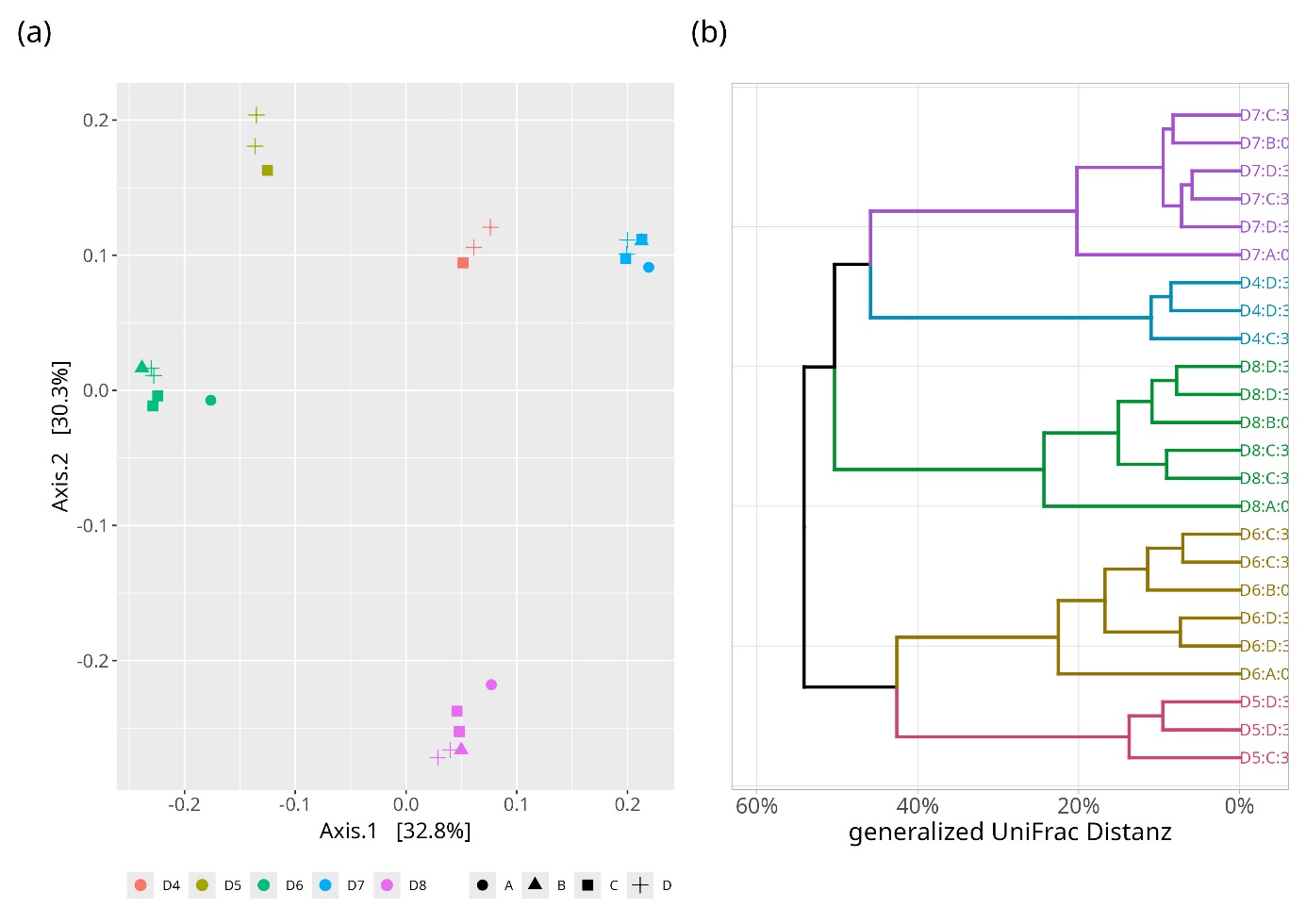


**Fig. S4** Beta diversity analysis based on generalized Unifrac distance


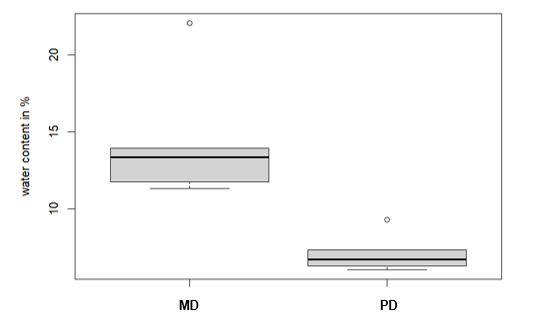


**Fig. S5** Boxplot of water content in % at time points t_4_ and t_5_, including median, minimum and maximum. MD: main-drying (primary); PD: post-drying (secondary)

**An example of a bacterial cell count report, through the QUANTOM Tx™ microbial cell counter:**


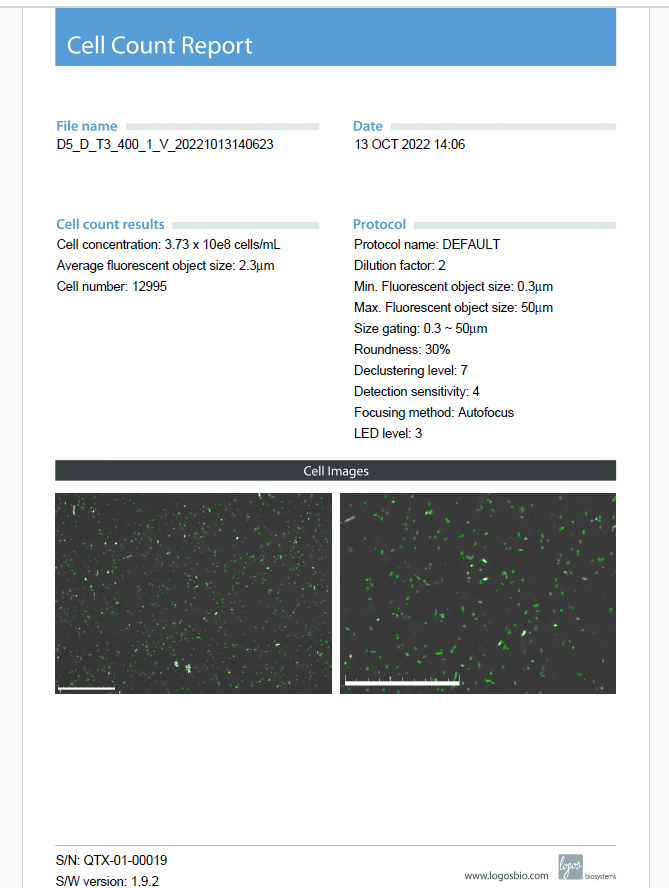


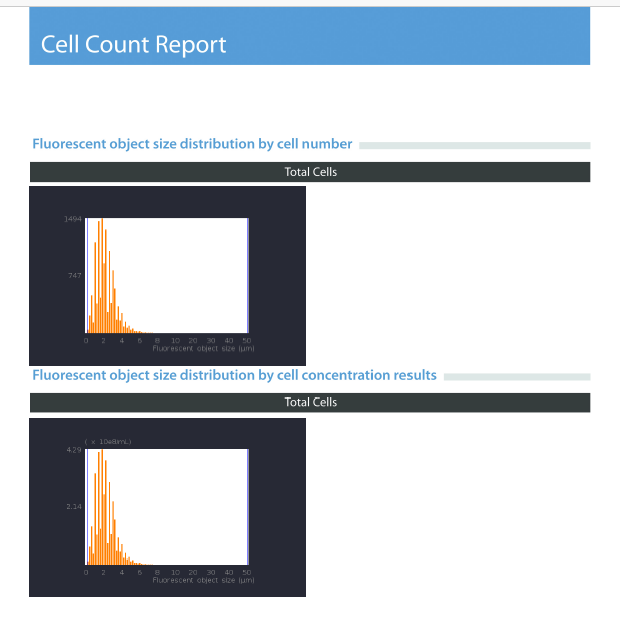


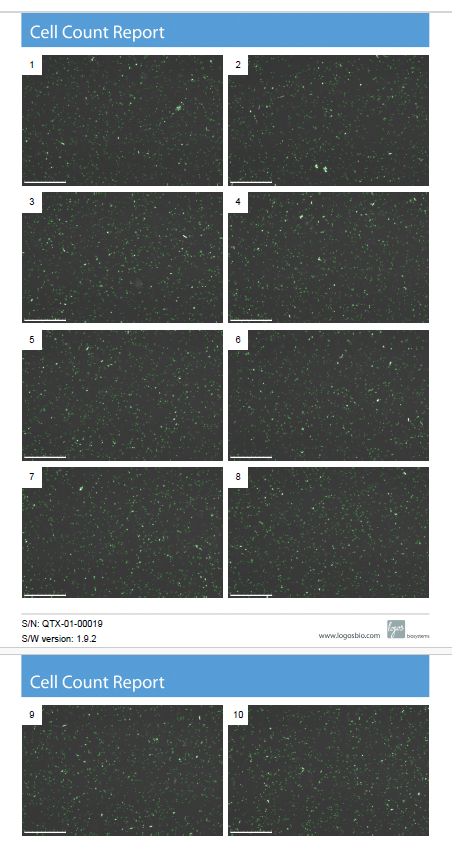

Supplement: Supplementary file 1 — Supplementary Material 1 [file 11274_2025_4291_MOESM1_ESM.docx]
